# Comparing machine learning models and traditional approaches for predicting obstructive coronary artery disease: a systematic review and meta-analysis

**DOI:** 10.3389/fcvm.2026.1892900

**Published:** 2026-07-30

**Authors:** Jingjing Guo, Chenggong Bao, Lihong Gong, Zhe Zhang

**Affiliations:** 1 The First Clinical College, Liaoning University of Traditional Chinese Medicine, Shenyang, Liaoning, China; 2 Key Laboratory of Ministry of Education for TCM Viscera-State Theory and Applications, Liaoning University of Traditional Chinese Medicine, Shenyang, Liaoning, China; 3 Key Research Office of the State Central Management Bureau on the Treatment of Sputum and Stasis Diseases, Affiliated Hospital of Liaoning University of Traditional Chinese Medicine, Shenyang, Liaoning, China

**Keywords:** coronary artery disease, machine learning, meta-analysis, obstructive coronary artery disease, systematic review

## Abstract

**Background:**

Obstructive coronary artery disease (oCAD) is a major cause of cardiovascular morbidity and mortality, and accurate prediction is essential for guiding clinical decision-making and secondary prevention. However, the potential of machine learning (ML) models for predicting oCAD remains insufficiently explored; therefore, this study aimed to compare the performance of ML methods with traditional approaches and evaluate differences among ML algorithm classes.

**Methods:**

A systematic search of EMBASE, Web of Science, Cochrane Library, Scopus, and PubMed was conducted from inception to February 9, 2026. Risk of bias was assessed using the Prediction Model Risk of Bias Assessment Tool. Random-effects meta-analysis was performed to pool AUCs of ML algorithms and compare the predictive performance of ML models with traditional approaches for oCAD. Meta-regression and subgroup analyses were used to explore heterogeneity.

**Results:**

A total of 41 studies were included in the review and meta-analysis. ML models showed significantly higher predictive performance than traditional approaches in 12 studies, although with substantial heterogeneity (MD: 0.09, 95% CI: 0.05–0.13; *p* < 0.00001; *I*^2^ = 95%). Heterogeneity in AUC was mainly associated with differences in diagnostic methods and predictors for oCAD. Among the algorithms evaluated, LR, RF, and XGBoost were the most commonly employed, suggesting their potential in clinical practice.

**Conclusions:**

ML shows promising discrimination relative to traditional scores. Future studies should emphasize adequate sample-size calculation, appropriate feature-selection strategies, and standardized handling of missing data and focus on developing and validating ML models based on accessible and noninvasive data sources.

## Introduction

Despite substantial medical advancements, coronary artery disease (CAD) remains the leading cause of mortality and a major global disease burden, of which ischaemic heart disease was one of the top causes of disability-adjusted life years among people aged 50–74 and 75+ years in 2019 (1). Earlier and more confident recognition of CAD in asymptomatic patients can improve secondary prevention, and noninvasive approaches for identifying obstructive coronary artery disease (oCAD) across all chest pain evaluation settings may enhance diagnostic accuracy, triage, and prevention. Although invasive coronary angiography (ICA) with functional flow measurements is essential for diagnosing oCAD, its diagnostic yield is as low as 23%–40% (2, 3), and the procedure is associated with risks, costs, radiation exposure, and discomfort, highlighting the need for first-line gatekeeper tools to guide its use (4, 5). Traditional approaches, such as the Framingham Risk Score (FRS) (6) and Diamond–Forrester (DF) model (7), are widely used to stratify the probability of oCAD. Other models incorporate additional variables such as high-sensitive troponin and C-reactive protein levels to enhance oCAD prediction (8, 9). However, knowledge and the availability of tools to predict the presence or absence of CAD in individual patients are limited, despite the emphasis on a patient-tailored approach in predictive, preventive, personalized medicine by using readily available patient data (10, 11).

The advent of machine learning (ML) has facilitated the development of data-driven models based on pattern extraction from large datasets, and this approach has been successfully applied to predict various cardiovascular outcomes (12, 13). ML can enhance the diagnosis of oCAD by integrating complex clinical information (14, 15). ML-based applications can potentially improve diagnostic accuracy, reduce costs and time, and support physicians in clinical decision-making. Previous systematic reviews have explored the effectiveness of ML in predicting heart failure, post-surgical mortality, atrial fibrillation, and other related conditions. However, the prediction of oCAD poses distinct clinical challenges (16). OCAD prediction is an immediate diagnostic task rather than long-term risk stratification, requiring high performance at the point of care. Because the diagnostic gold standard is invasive and costly, accurate noninvasive models are essential. Moreover, the predictive features are highly heterogeneous, ranging from clinical risk factors and electrocardiography (ECG) parameters to advanced imaging characteristics. Therefore, this study systematically evaluated the application of ML in oCAD prediction and evaluated its differences from traditional cardiovascular risk assessment.

## Methods

This study was performed in accordance with the Preferred Reporting Items for Systematic Reviews and Meta-Analysis (PRISMA) statement (17) and the Checklist for Critical Appraisal and Data Extraction for Systematic Reviews of Prediction Modeling Studies (CHARMS) (18). This review did not require ethical approval, since it was a systematic review and meta-analysis.

### Search strategy

The EMBASE, Web of Science, Cochrane, SCOPUS, and PubMed databases were searched from inception through February 9, 2026. Search items were designed on the basis of the combination of medical subject headings and free words: “Obstruct*,” “Coronary,” “Coronary heart disease,” “Myocardial Ischemia,” “machine learning,” “Learning, Machine,” “Transfer Learning,” “Artificial Intelligence,” “AI,” “Artificial Intelligence.” The search strategy is detailed in Supplementary Table S1.

### Inclusion and exclusion criteria

We selected studies that (1) constructed a complete predictive model, (2) presented the results of ML algorithms, (3) were published in English and (4) involved patients with oCAD. Exclusion criteria were as follows: (1) studies with incomplete data; (2) case reports, conference abstracts, reviews, letters, patents.

### Study selection

All articles identified were imported into EndNote X9. First, the titles and abstracts of the articles were reviewed after removing duplicates, and the papers that did not meet the inclusion criteria were excluded. Subsequently, the full texts of the remaining articles were reviewed, and only the papers that met the inclusion criteria were included. If the title or abstract did not provide sufficient information to determine a paper's eligibility, we reserved the paper for the next phase. Two reviewers independently conducted the literature search and screening, and disagreements were resolved by a third reviewer.

### Data extraction

Three researchers independently extracted the following data from the included studies: the first author, publication year, nation, study design, age, sex ratio, sample size, number of events, c-index [area under the curve (AUC)], 95% confidence intervals (CIs) of the c-index, type of algorithm, diagnosis and predictors. We characterized the dataset partitions of each study as training, internal validation, and external validation. For studies that reported AUC values of both internal and external validation cohorts, data from the latter were abstracted for analysis (18–20).

### Outcomes

The primary outcome assessed in this study was the comparative discrimination between ML models and traditional approaches in predicting oCAD. The AUC was selected as the primary measure of discrimination, since it reflects overall model accuracy without requiring a predefined diagnostic threshold (21). For the secondary outcome, we pooled the AUC values of commonly used ML algorithms across the included studies.

### Risk of bias

The risk of bias for each model was assessed using the Prediction Model Risk of Bias Assessment Tool (PROBAST) (22). Three reviewers conducted the assessments independently, resolving discrepancies through discussion.

Based on these assessments, we performed a sensitivity analysis excluding high-risk-of-bias studies per PROBAST. Performance of ML vs. traditional approaches, and across ML models, was reassessed using the same framework as the main analysis, restricted to low-risk-of-bias studies.

### Statistical analyses

Studies comparing ML models with traditional approaches were analyzed using Stata 12.0 (Stata Corp, College Station, TX, USA). Mean difference (MD) and weighted mean difference (WMD) were used as the effect measures in the meta-analysis (23). MD was calculated as AUC (ML)—AUC (traditional). For studies reporting AUC with 95% CI values, the standard deviation (SD) was calculated using RevMan calculator (19, 24). Similar to previous meta-analyses, when studies reported multiple ML models derived from the same dataset, the model with the highest AUC was selected to avoid unit-of-analysis errors (25–28). Single-arm meta-analyses were conducted in R Studio version 2024.09.1 (R Studio, PBC) running R version 4.4.2, which was also used to generate forest plots. Random-effects models were applied throughout to account for statistical heterogeneity. We quantified the heterogeneity using *I*^2^ values, with *I*^2^ values of 25%, 50%, and 75% set as the cut-offs for low, moderate, and high heterogeneity, respectively (29). All results were reported as mean with 95% CI (lower limit, upper limit).

If heterogeneity was observed, exploratory meta-regression was conducted to explore the potential source of variability in prevalence estimates across studies. Meta-regression can simultaneously explore the effects of multiple study characteristics on the variance of pooled estimates (30). All findings were interpreted with caution due to multiple comparisons. The meta-regression command in Stata 12.0 (Stata Corp, College Station, TX, USA) was used to model the adjusted association between multiple explanatory variables and the AUC of the model. On the basis of previous evidence, we considered the following study characteristics to be potential sources of heterogeneity: country of publication, type of ML model, study design, AUCs for the test and external validation cohorts, predictors and diagnostic criteria for oCAD (Supplementary Table S2). Subgroup analyses were conducted to further investigate the sources of heterogeneity. Details of the subgroup classification are provided in Supplementary Table S2.

## Results

### Results of the search

**Figure 1:**
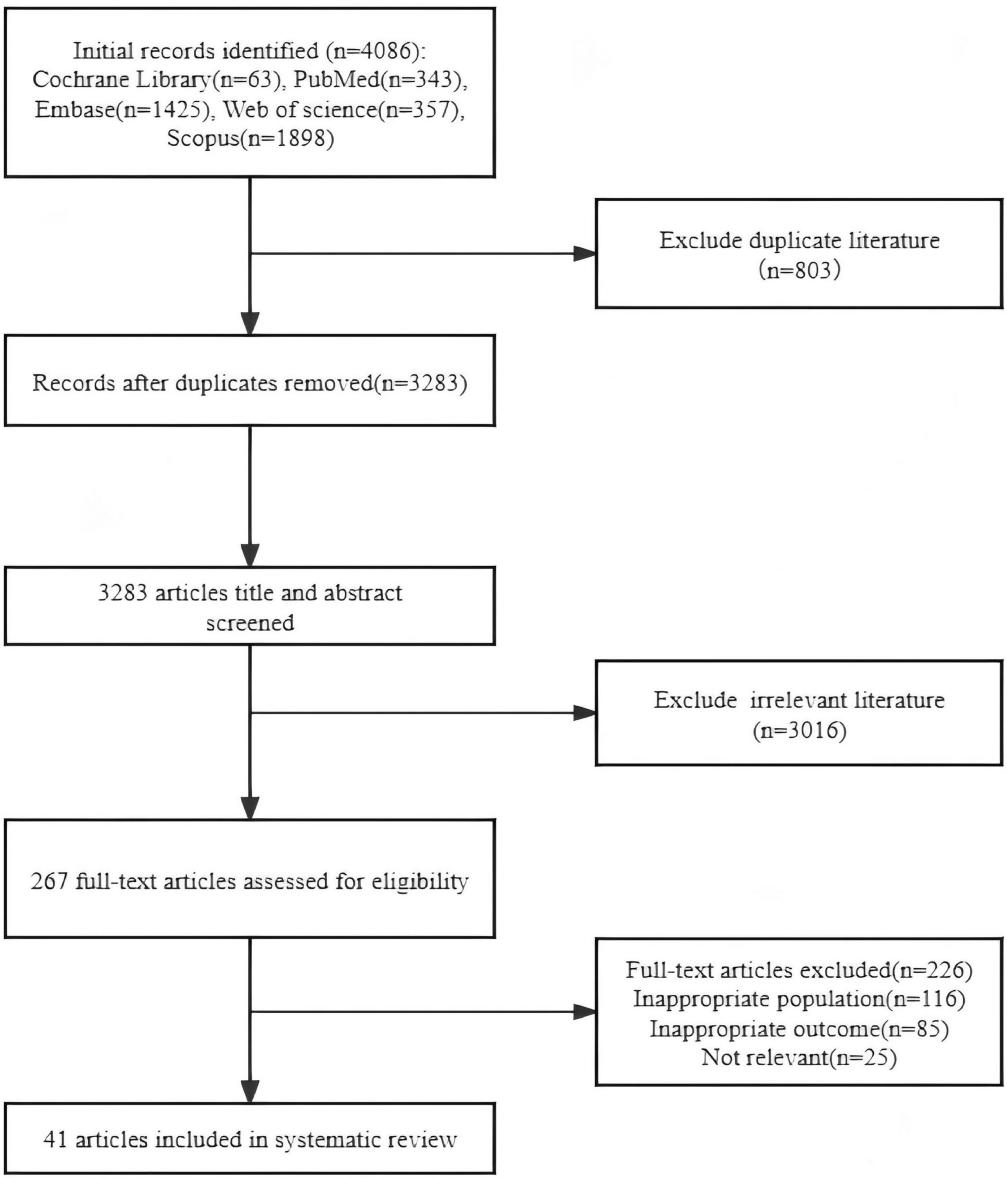
Flow diagram of study selection.

In total, our systematic search yielded 4,086 records. After removing 803 duplicate records, a total of 3,283 records remained for title and abstract screening. After applying our exclusion criteria, we excluded 3,016 studies and retrieved full-text articles for 267. After a thorough full-text review, 226 articles were further excluded, leaving 41 studies for data abstraction (Figure 1).

### Included studies

**Figure 2:**
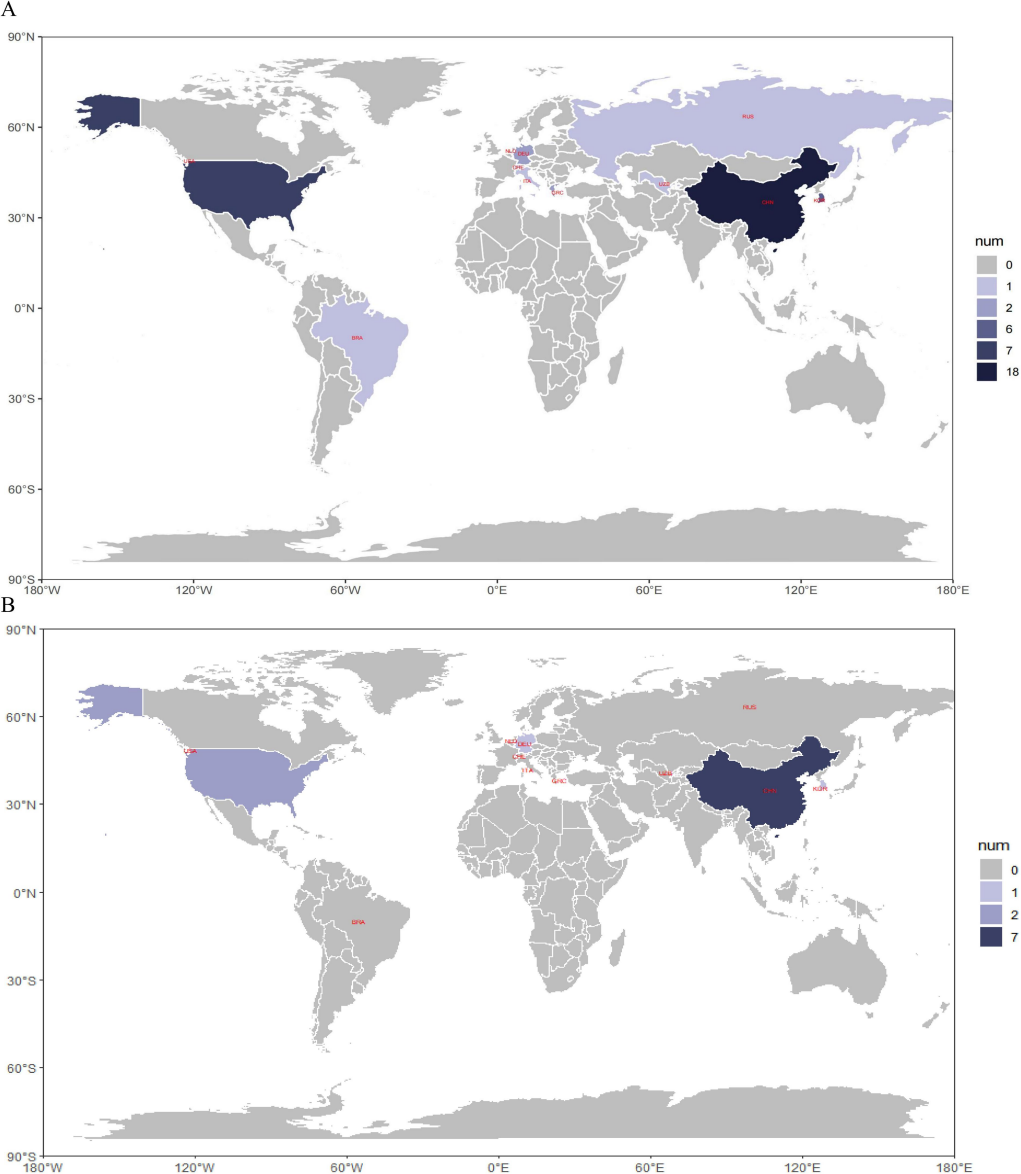
Global geographical distribution of the included studies. **(A)** Distribution of all studies included in this meta-analysis. **(B)** Distribution of countries that conducted external validation. BRA, Brazil, CHN, China; DEU, Germany; GRC, Greece; ITA, Italy; NLD, Netherlands; RUS, Russia; KOR, South Korea; CHE, Switzerland; USA, USA; UZB, Uzbekistan.

The publication dates of the 41 studies included for data abstraction ranged from 2018 to 2026. These 41 studies encompassed 11 countries (16, 31–56) and nine models. The algorithms assessed in these studies included extreme gradient boosting (XGBoost) (*n* = 16) (31, 34, 36, 37, 39–44, 54, 57–61), light gradient-boosting machine (LightGBM) (*n* = 2) (31, 41), random forest (RF) (*n* = 17) (31, 34, 35, 41, 43, 44, 48, 50, 53–55, 57, 58, 62–65), multilayer perceptron (MLP) (*n* = 5) (31, 41, 64–66), support vector machine (SVM) (*n* = 10) (41, 43, 46, 50, 52, 54, 55, 58, 64, 65), deep learning (DL) (*n* = 12) (32, 33, 35, 38, 45, 49, 65, 67–71), k-nearest neighbors (KNN) (*n* = 5) (34, 41, 43, 50, 64), logistic regression (LR) (*n* = 15) (31, 34, 35, 43, 44, 46, 50–54, 56, 57, 63, 65) and CatBoost (*n* = 2) (41, 58). Only 12 studies (33, 39, 41, 42, 47–51, 57, 67, 70) that reported data comparing the predictive ability of ML models with traditional approaches were included in a two-arm meta-analysis. Details of each study, including patient age and BMI, diagnostic criteria, regions, and traditional approaches are provided in Supplementary Table S3, Table 1 and Figure 2.

**Table 1:** Detailed comparison of traditional approachs and ML algorithm in included studies.

Study	ML Algorithm	Traditional approach
Aikeliyaer Ainiwaer	DL	The Diamond-Forrester score
Byoung Geol Choi	LR	The CAD consortium clinical score
Guanhua Dou	XGBoost	The PTP model (European Society of Cardiology)
J. A. van Dalen	XGBoost	The expert readers
JoonNyung Heo	LR	The Framingham Risk Score
Juntae Kim	XGBoost	The CAD consortium clinical score
Ling-yun Zhou	RF	Duke clinical score
Lohendran Baskaran	XGBoost	The CAD consortium clinical score
Michael J. Zellweger	The optimized MPA model	The Framingham Risk Score
Parastoo Dehkordi	DL	The AHA Pre-Test Probability Model
Rishi K. Trivedi	DL	The CAD consortium clinical score
Subhi J. Al’Aref	XGBoost	The CAD consortium clinical score

### Meta-analysis comparing the predictive ability of ML models vs. traditional approaches.

**Figure 3:**
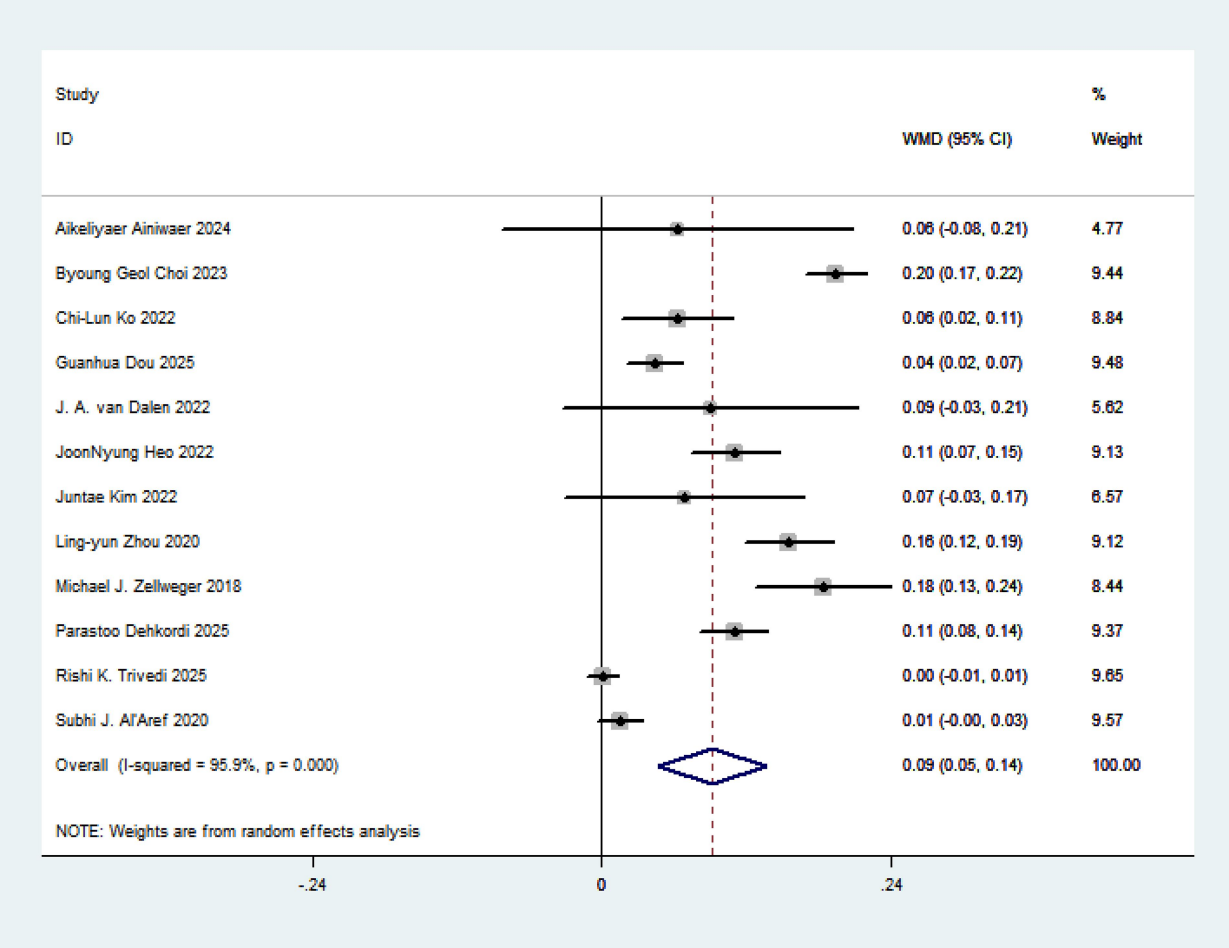
Predictive performance of machine learning models versus traditional approaches for obstructive coronary artery disease (oCAD).

From our pooled analysis of 12 studies with 89,143 patients, we observed a significantly higher mean AUC for ML models than for traditional approaches in the same populations [MD: 0.09, 95% CI: (0.05, 0.13), *p* < 0.00001, *I*^2^: 95%; Figure 3].

**Figure 4:**
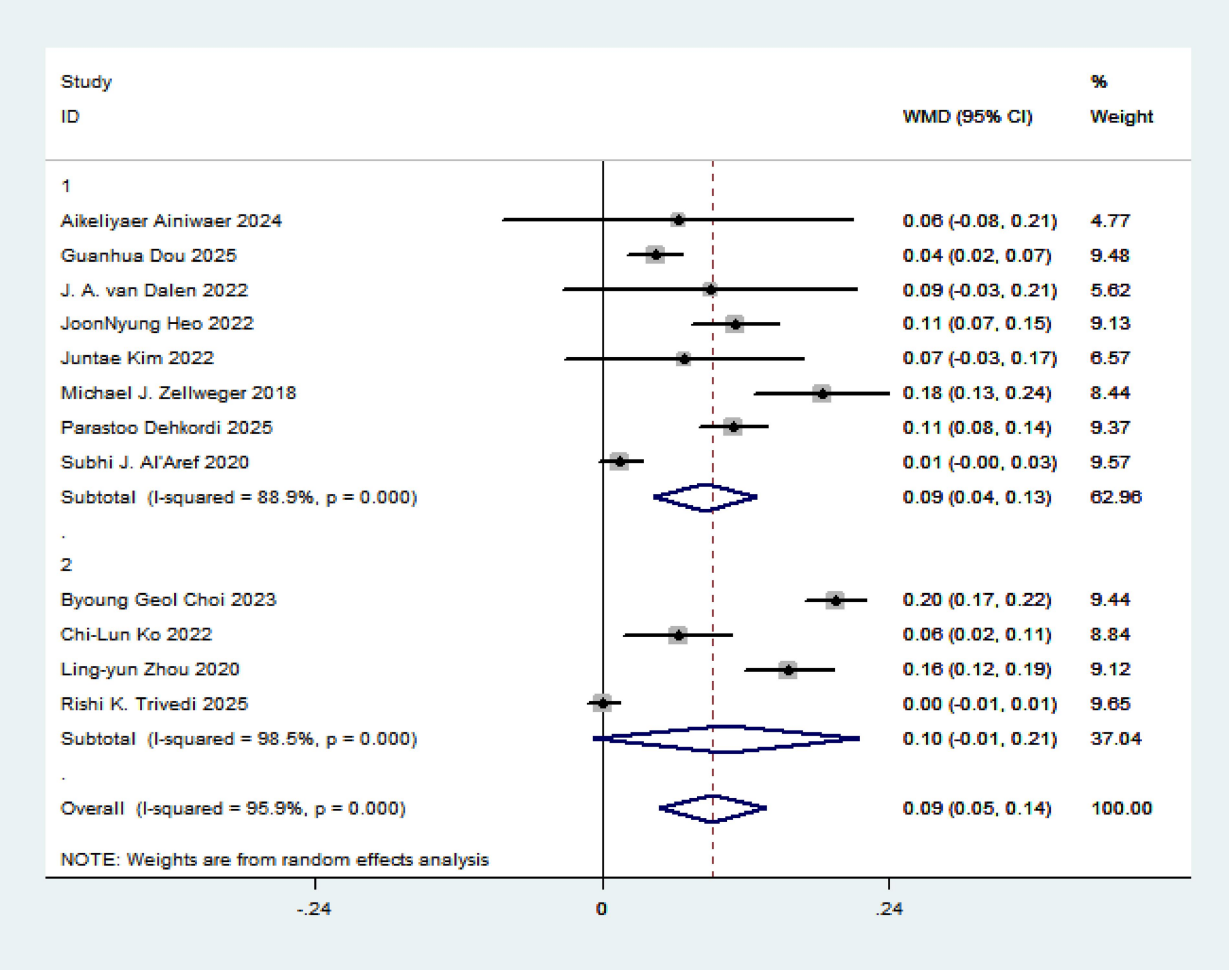
Subgroup analysis of predictive performance based on external validation status. Comparison of machine learning models and traditional approaches for predicting oCAD, stratified by whether external validation was performed. (1) Studies without external validation; (2) Studies with external validation.

The subgroup analysis showed that in the test group, the pooled WMD was 0.08 (95% CI: 0.05–0.12), with significant heterogeneity (*I*^2^ = 84.6%, *p* < 0.001). In the external validation group, the pooled WMD was 0.10 (95% CI: 0.01–0.21), also with significant heterogeneity (*I*^2^ = 98.5%, *p* < 0.001; Figure 4).

We performed meta-regression to explore the potential sources of the considerable heterogeneity in the studies. The results indicated that the source of heterogeneity in the studies was probably associated with predictors and diagnostic criteria for oCAD, as illustrated in Table 2.

**Table 2:** Results of meta-regression analysis for predicting oCAD.

Covariates	*β*	95% CI	*p*-Value
Country of publication	0.001	[−0.019, 0.020]	0.951
Diagnosis	−0.03	[−0.060, 0.001]	0.054
External verification	0.017	[−0.060, 0.095]	0.66
Predictors	−0.045	[−0.083, −0.007]	0.021
Study design	−0.012	[−0.095, 0.071]	0.781
Type of ML model	0.002	[−0.022, 0.026]	0.857

**Figure 5:**
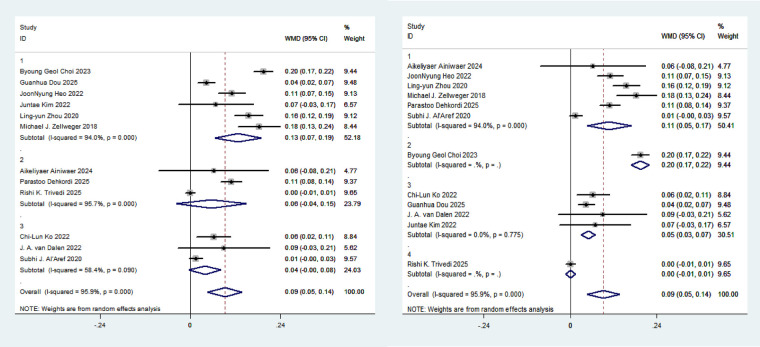
Subgroup analysis of machine learning models versus traditional approaches for predicting °CAD. Studies were stratified by: **(A)** predictors. 1: clinical features only; 2: biological signals (± clinical features); 3: imaging-based predictors (± clinical features); and **(B)** diagnostic thresholds for oCAD. 1: stenosis ≥50%; 2: stenosis; 3:≥70% stenosis in major branches or ≥50% in left main artery; 4: PC1 performed based on interventional cardiologists' assessment. Detailed diagnostic criteria are available in Supplementary Table S2.

Subgroup analysis based on predictor type demonstrated that the pooled effect size differed across groups. The pooled WMD was 0.13 (95% CI: 0.07–0.19) for clinical features, 0.06 (95% CI: −0.04 to 0.15) for biological signals, and 0.04 (95% CI: 0.01–0.07) for imaging-based predictors. Significant heterogeneity was observed in the first two subgroups, whereas heterogeneity was low in the imaging-based predictors (*I*^2^ = 29.5%) (Figure 5A).

Subgroup analysis by diagnosis showed pooled WMDs of 0.11 (95% CI: 0.05–0.16) for ≥50% stenosis (group1), 0.20 (95% CI: 0.17–0.22) for >70% stenosis (group2), 0.05 (95% CI: 0.03–0.07) for oCAD, defined as left main stenosis greater than 50% or stenosis greater than 70% in any other significant vessels (group3), and 0.00 (95% CI: −0.01 to 0.01) for post-PCI patients (group4), with varying degrees of heterogeneity across subgroups (Figure 5B).

**Figure 6:**
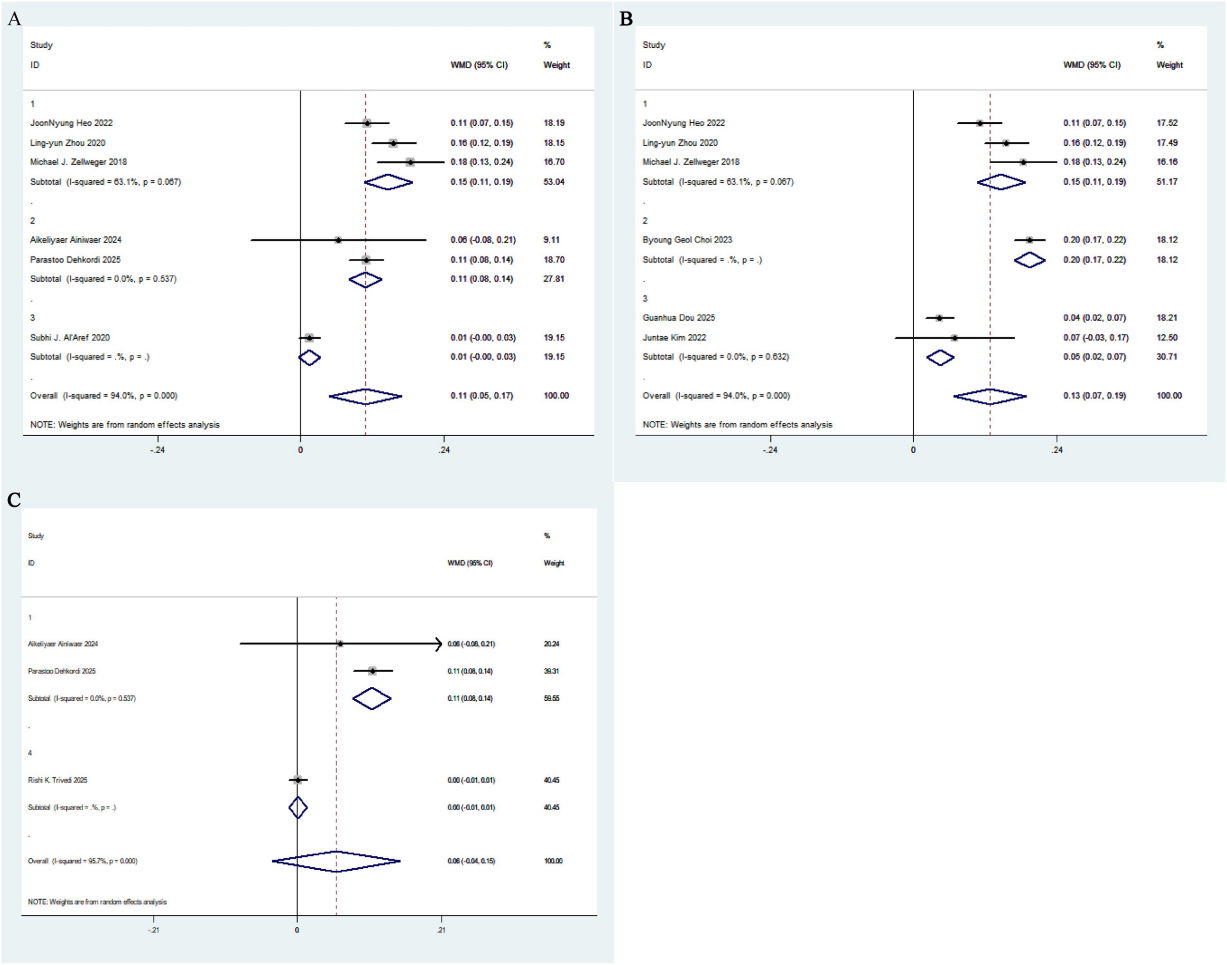
Subgroup analyses for heterogeneity. Forest plots showing results of re-analyses for subgroups with high heterogeneity. **(A)** Diagnosis subgroup re-stratified by predictors. **(B)** Predictors subgroup 1 re-stratified by oCAD diagnostic criteria. **(C)** Predictors subgroup 2 re-stratified by oCAD diagnostic criteria. Detailed diagnostic criteria are available in Supplementary Table S2.

Owing to the high heterogeneity in the clinical and biological signal predictor subgroups, additional subgroup analyses were performed on the basis of diagnostic categories. In the clinical features group, the pooled WMD was 0.15 (95% CI: 0.11–0.19) for group1 and 0.05 (95% CI: 0.02–0.07) for group3. In the imaging-based predictors group, the pooled WMD was 0.11 (95% CI: 0.08–0.14) for group1. Heterogeneity decreased after subgroup analysis (Figure 6).

### Meta-analysis of pooled AUC means

**Figure 7:**
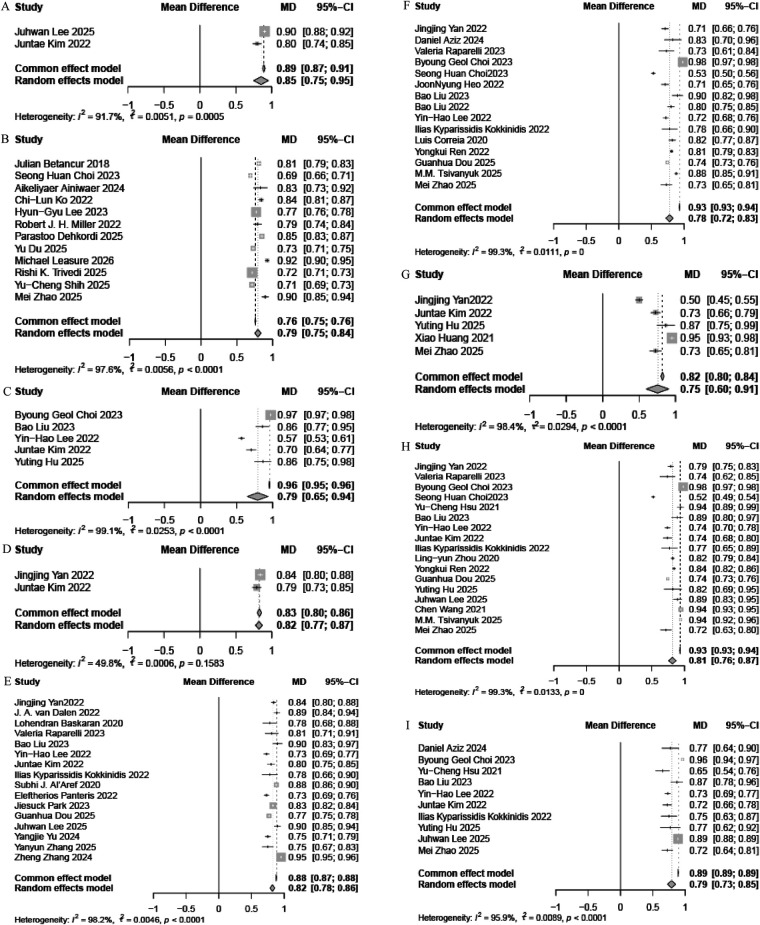
Performance comparison of machine learning models. **(A)** CatBoost; **(B)** DL; **(C)** KNN; **(D)** LightGBM; **(E)** XGBoost; **(F)** LR; (0) MLP; b RF; **(1)** SVM. CatBoost. Categorical Boosting; DL, deep learning; KNN, K-nearest neighbors; LightGBM, Light Gradient Boosting Machine; LR, logistic regression; MLP, multilayer perceptron; RF, random forest; SVM, support vector machine; and XGBoost. Extreme Gradient Boosting.

The analysis was restricted to data from the test and the external validation sets. The pooled mean AUC values of 12 studies reporting the DL algorithm was 0.79 (95% CI: 0.75, 0.84, *I*^2^: 97.6%). For the KNN algorithm, the pooled AUC from five studies was 0.79 (95% CI: 0.65, 0.94, *I*^2^: 99.1%). Two studies evaluating the LightGBM algorithm had a mean AUC value of 0.82 (95% CI: 0.77, 0.87, *I*^2^: 49.8%); 15 studies evaluating the LR algorithm had a mean AUC value of 0.78 (95% CI: 0.72, 0.83, *I*^2^: 99.3%); five studies evaluating the MLP algorithm had a mean AUC value of 0.75 (95% CI: 0.60, 0.91, *I*^2^: 98.4%); 17 studies evaluating the RF algorithm had a mean AUC value of 0.81 (95% CI: 0.76, 0.87, *I*^2^: 99.3%); 10 studies evaluating the SVM algorithm had a mean AUC value of 0.79 (95% CI: 0.73, 0.85, *I*^2^: 95.9%); 2 studies evaluating the CatBoost algorithm had a mean AUC value of 0.85 (95% CI: 0.75, 0.95, *I*^2^: 91.7%);and 16 studies evaluating the XGBoost algorithm had a mean AUC value of 0.82 (95% CI: 0.78, 0.86, *I*^2^: 98.2%)(Figure 7). Considering the high between-study heterogeneity, subgroup analyses stratified by diagnosis and predictor were further conducted, and the results are summarized in Supplementary Figures S1–S7.

### Risk of bias

**Figure 8:**
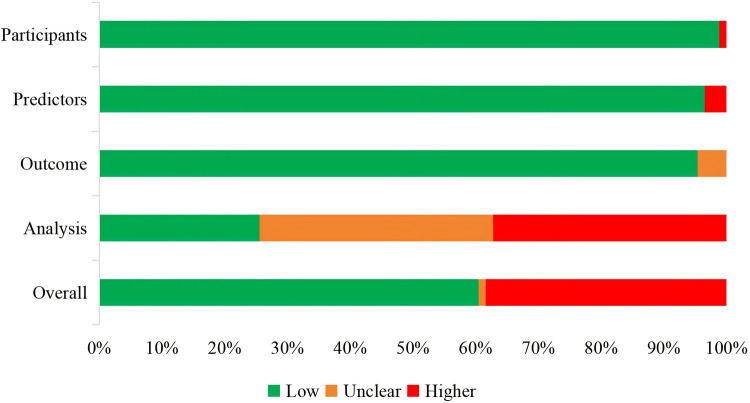
PROBAST risk of bias assessment summary for machine learning models in the included studies.

Using the PROBAST tool, 33 out of 86 ML models (38.4%) across 41 studies demonstrated an overall high risk of bias (Figure 8). Participant selection was a concern in 1 models (1.16%). Selection and use of predictors and outcomes were of concern in 3 (3.49%) and 4 (4.65%) models, respectively. Analysis was of greatest concern in 64 (74.42%) models because of poor reporting of discrimination and calibration statistics or inappropriate handling of missing data.

The sensitivity analysis restricted to low-risk-of-bias studies yielded results broadly consistent with the main analysis. Of note, KNN showed notable improvement in the low-risk-of-bias cohort, whereas performance of other ML algorithms remained largely stable (Supplementary Figures S8–S9).

## Discussion

After accounting for substantial heterogeneity through subgroup analysis, which could be primarily attributed to variations in predictors and diagnosis, our findings indicate that ML models hold promise in assisting clinicians with the prediction of oCAD. Among the algorithms evaluated, LR, RF, and XGBoost were the most commonly employed, suggesting their potential in clinical practice. Notably, any apparent performance advantage is highly context-dependent and varies by diagnostic threshold, input modality, and study quality.

Ischemic heart disease can be categorized into oCAD, which results from the rupture or erosion of atherosclerotic plaques, and non-obstructive CAD, which arises from impaired arteriolar vasodilation, and leads to an inadequate increase in blood flow during stress than during rest (72, 73). According to the 2024 ESC Guidelines for the Management of Chronic Coronary Syndromes, oCAD is defined as a coronary stenosis rate of ≥50% in the left main coronary artery (LM) or ≥70% in other major epicardial vessels (74). Patients with oCAD generally present with a higher baseline cardiovascular risk, and in comparison with non-oCAD patients, individuals with oCAD also face a two- to three-fold greater risk of death or major cardiovascular events (73). The 2024 ESC Guidelines updated the pre-test probability [risk factor-weighted clinical likelihood (RF-CL)] model, which incorporates age, sex, symptom characteristics, and the number of risk factors to precisely predict the likelihood of oCAD, guiding clinicians in selecting appropriate diagnostic and therapeutic strategies (74). Nevertheless, accurately distinguishing patients with oCAD from those with atypical presentations remains a major challenge for cardiologists (53).

The 2023 ACC/AHA guideline and 2024 ESC Guidelines have emphasized the importance of initial risk stratification to avoid unnecessary diagnostic procedures in patients with suspected CAD (74, 75). The 2012 guideline recommended the Duke Clinical Score (DCS) and the Diamond-Forrester Model (DFM) as the preferred tools for this purpose (76). In a study by Baskaran et al., the CAD2 model, which incorporates both traditional risk factors and lipid panel data, demonstrated improved discrimination and reclassification abilities than the Coronary Risk Score (CRS) and the Updated Diamond-Forrester (UDF) model during initial clinical assessment of patients presenting with chest pain (77). Implementing more accurate models in frontline evaluation could improve the efficiency of diagnosing oCAD. With the advent of artificial intelligence, ML algorithms have become capable of capturing complex, non-linear interactions among variables (78, 79). The 2024 ESC Guidelines recommended the RF-CL model, which incorporates symptoms and the number of risk factors to improve the prediction of oCAD (74). ML is reshaping downstream clinical decision-making pathways by enhancing predictive transparency, dynamically integrating clinical workflows, and avoiding treatment paradoxes. These models can manage high-dimensional data and intricate relationships, ensuring that predictions for oCAD are no longer confined to traditional risk-scoring methods.

In previous meta-analyses comparing traditional approaches with ML models for predicting atherosclerotic cardiovascular risk and all-cause mortality after transcatheter aortic valve replacement, ML models showed potential predictive performance (19, 21). However, a meta-analysis by Zhoujian Sun et al. on the prediction of heart failure events found that ML models did not exhibit a significant advantage and were less clinically feasible and reliable than traditional methods (80). The authors attributed these limitations to factors such as insufficient data, poor data quality, and the absence of external validation. They highlighted the need to explore methods for extracting information from longitudinal sequential datasets to improve event prediction, an area particularly suited to ML algorithms. Unlike long-term prognostic modeling, this study systematically evaluated ML models in the context of the immediate diagnostic task of oCAD and explored factors contributing to the significant heterogeneity in pooled AUC estimates. Our findings suggest that, in comparison with traditional approaches, ML shows greater potential to assist clinicians in predicting oCAD. Similar to the findings of previous studies, our findings indicated that external validation often reveals performance degradation of models in real clinical settings, highlighting its indispensable role in assessing model robustness (81, 82). Our findings also suggest that using a unified and authoritative diagnostic standard for oCAD may reduce label noise and improve the signal-to-noise ratio, thereby enhancing AUC performance. Thus, high-quality labeling is an important determinant of model accuracy. We also observed marked heterogeneity in the features used across studies. Most models combined traditional clinical variables with advanced imaging parameters, indicating that performance gains may depend not only on algorithm optimization but also on the integration of multi-source information through feature engineering. Min-Young Yu et al. analyzed the heterogeneity observed in their study (83) and they observed a statistically significant negative association between sample size and AUC for ML-based models. Therefore, this “trend” is still worth noting, and with larger sample sizes and more rigorous validation designs, models may demonstrate more robust and better performance. With the ongoing advancements in artificial intelligence research, increasing attention is being paid to data quality, database development, and model validation. Our findings are consistent with the findings of most recent studies, highlighting the importance of these factors in enhancing the clinical utility of ML-based prediction models. However, owing to limitations in experimental methods, comparing the results with traditional methods by selecting the optimal AUC carries the risk of being overly optimistic.

The 12 head-to-head studies compared ML models with traditional methods for predicting obstructive CAD prior to invasive angiography. These models could be applied in two key clinical scenarios: pre-test probability estimation to identify low-risk patients who may safely avoid invasive procedures, and post-CTA triage to refine referral decisions for intermediate-risk patients. These ML models hold promise for reducing unnecessary invasive coronary angiography, though prospective validation is still needed. The integration of ML-based risk stratification into clinical workflows could optimize the utilization of downstream diagnostic tests. By accurately identifying patients with a very low probability of oCAD, ML models may safely defer the need for anatomical or functional assessments such as CTA, stress imaging, or invasive angiography. Conversely, for high-risk individuals, ML predictions could prioritize urgent invasive evaluation, thereby enhancing resource allocation and reducing overall healthcare costs.

ML was originally described as a program capable of learning to perform tasks or make decisions automatically on the basis of data (84). Its application in healthcare has been propelled by the growth of big data analytics and the increasing demand for evidence-based care (85). ML models are widely regarded as a revolutionary innovation with the potential to transform the entire healthcare system and have increasingly been utilized to develop prognostic prediction models (84, 86). XGBoost is an advanced ensemble learning algorithm that builds a strong predictive model by iteratively combining multiple weak models that typically represent short decision trees, each trained to correct the errors of its predecessors (87). LR and RF are two widely used ML algorithms that are commonly applied to both classification and regression tasks. LR is a linear model primarily employed for binary classification, while RF is an ensemble learning method that enhances generalization by constructing multiple decision trees and combining their outputs through voting or averaging (88). LightGBM is an efficient gradient-boosting decision tree framework based on Gradient-based One-Side Sampling (GOSS) and Exclusive Feature Bundling (EFB), which can increase training speed by over 20 times while ensuring high accuracy (89).

This systematic review and meta-analysis showed that LR, RF, and XGBoost were the most commonly employed algorithms, suggesting their potential for clinical practice. The XGBoost algorithm has been increasingly applied in cardiovascular healthcare, and it has shown superior predictive accuracy in comparison with traditional approaches (87). An analysis of the NCDR database showed that an XGBoost algorithm predicted major bleeding after percutaneous coronary intervention with a c-index of 0.82, outperforming the existing NCDR model, which had a c-index of 0.78 (90). In a single-center study involving 11,190 patients undergoing index cardiac surgeries, Arman Kilic et al. showed that XGBoost provided modest yet statistically significant improvements over the STS risk models in predicting operative mortality (91). Our findings indicated that RF and LR models were also commonly used, and their AUC values were similar; however, their performance was not as favorable as that of XGBoost. In a longitudinal cohort study focused on cardiovascular disease, the differences among the three algorithms were minimal, with LR demonstrating only a slight advantage (92). As noted by Arman Kilic, ML may serve as a complementary tool in predictive analytics, since certain patients and outcomes may be more accurately predicted by specific ML algorithms, while others may be better suited to traditional models such as logistic regression (87). The CatBoost algorithm demonstrates superior performance and higher heterogeneity, while the LightGBM algorithm demonstrates superior performance and lower heterogeneity, but both have been utilized in relatively few studies to date. Additional research is necessary to validate their effectiveness in predicting oCAD. The results of our analyses indicate minimal differences in predictive performance among conventional ML models such as DL, KNN and SVM. In comparison, the DL, KNN, and SVM algorithms achieved comparatively low AUC values, with the MLP algorithm achieving the lowest AUC across all algorithms. Notably, any apparent performance advantage is highly context-dependent and varies by diagnostic threshold, input modality, and study quality.

The geographic distribution of included studies, with predominant representation from China, the USA, and South Korea, reflects regional differences in ML research adoption. The absence of studies from many regions highlights the need for broader international collaboration and external validation across diverse populations to ensure global applicability of these models before widespread clinical implementation. The PROBAST assessment indicated that the main issues in the ML meta-analysis are related to the analysis methodology. First, sample-size calculations were not performed, and some studies had insufficient sample sizes. Second, handling of missing data was inadequate, with some studies opting to simply delete missing values. Third, feature selection was based solely on univariate regression, which may have overlooked important interactions. Lastly, the weights of the features used in the ML models were not reported, nor were the results consistent with the reported outcomes of multivariable analysis. These factors may have influenced the reliability and interpretability of the findings. In addition, the marked improvement of KNN in the low-risk-of-bias cohort suggests that this algorithm may be more sensitive to study quality than other ML models.

### Strengths and limitations of this study

A major strength of this study was that it performed a comprehensive evaluation of both the clinical feasibility and reliability of traditional approaches and ML models and explored potential sources of heterogeneity. Another key strength was the focus on assessing the predictive accuracy of commonly used ML models. However, this study also had several limitations: (1) We did not conduct a calibration analysis, since most studies either failed to report calibration metrics or used inappropriate calibration methods. (2) The study focused exclusively on predictive accuracy and did not explore the risk factors associated with oCAD. (3) Selecting the highest AUC model from each study may introduce selection bias, potentially overestimating ML performance.

## Conclusion

Our findings suggest that ML shows promising discrimination relative to traditional scores. Among the algorithms evaluated, LR, RF, and XGBoost were the most commonly employed, suggesting their potential in clinical practice. Notably, any apparent performance advantage is highly context-dependent and varies by diagnostic threshold, input modality, and study quality. However, strong external validation, careful calibration, standardized diagnostic criteria, and assessment of net clinical benefit are needed before these models can be adopted in routine practice. Methodological appraisal indicated that future studies should emphasize adequate sample-size calculation, appropriate feature-selection strategies, and standardized handling of missing data. Further studies should focus on developing and validating ML models based on accessible and noninvasive data sources.

## Data Availability

The original contributions presented in the study are included in the article/Supplementary Material, further inquiries can be directed to the corresponding author.
